# Parliamentary Bills and Returns

**Published:** 1855

**Authors:** 


### PARLIAMENTARY BILLS AND RETURNS.

### I. *Churchyards in London.*

From a return made to the House of Commons of all the notices for discontinuance of burials within the metropolis, bearing date March 7th, 1854, it appears that from January 19th, 1850, to March 2nd, 1852, no fewer than 242 grave-yards were ordered to be closed in the course of the years 1853–59. In the majority of cases, the closure was ordered to take place forthwith; in a few cases, one or two years were allowed In some instances, the vaults only of the burial-places were ordered to be closed immediately, the open part being permitted to receive the dead for brief periods longer. The Holy Trinity burial-ground, in Brompton, has received the longest respite; it remains open to the 1st of October, 1859: its vaults, however, are closed. The All Souls Roman Catholic cemetery at Chelsea is to remain open until the 23rd of August, 1858. In cases where a part of a graveyard has not been at all used, the unused portion is permitted to remain open for a limited time, an intimation being given that only one body is to be deposited in one grave.

These peremptory orders in relation to the removal of grave-yard nuisances, are but the consummation of the indefatigable and almost unrecognized labours of Mr. Walker, the distinguished author of "Gatherings from Grave-yards." It is plain that the mere closing of a grave-yard is the merest temporary amelioration of mischief. It leads, however, directly to the greater question, "What shall be done with the dead?"—a question which we shall freely discuss on another occasion.

---

### II. *Metropolis Drainage.*

In relation to this important question, we learn from a letter, dated the 21st day of July, from Mr. Morewood, to her Majesty's Secretary of State for the Home Department, in reference to two tunnel sewers for the arterial drainage of the metropolis, the following facts:—

"Islington, London, 21st July 1854.

"1. That 70,000,000 gallons of water are daily raised to the surface of a densely-inhabited valley, and there converted into sewage, stagnating in the low-lying districts of the metropolis, and daily polluting the atmosphere and the Thames, they urgently demand an efficient outfall.

"2. That the metropolitan commission of sewers do not propose the removal of the injury resulting to the inhabitants, but to spend 1,050,000*l.* in covering over a naturally-formed rivulet, meandering through open fields, "the Hackney Brook," and in diverting similar rural drainage into the populous locality of Deptford.

"3. That the Great London Drainage Plan of two tunnel sewers running through the most populous localities, extending from Chelsea and Vauxhall respectively to the marshes east of the metropolis would bc capable of removing not only 70,000,000, but 270,000,000 gallons of sewage daily, and would cost 1,000,000*l.*

"4. That this plan rests upon actual surveys and levels, confirmed estimates, borings, and data collected during a series of years. Its having been prosecuted through the agency of commercial enterprise, as the only means available to private individuals, and its having been twice submitted to the scrutiny of a select committee, is respectfully urged in its behalf.

(Signed) "J. J. Morewood."

---

### III. *Metropolis Water.*

From an abstract of a return made to the House of Commons on the 1st of June 1854, the subject of the average number of gallons of water supplied daily by each of the several metropolitan water companies, during the twelve months ending the 31st day of December 1853, and stating whether the supply can be increased, or is in course of being increased, together with the number of houses, manufactories, and public establishments supplied with water by each company, we gather the following results:—

### New River.

The average number of gallons of water supplied daily by the New River Company to houses, manufactories, and public establishments in the year 1853 was 17,537,396, and this quantity is capable of being increased to 29,000,000.

### East London Waterworks.

The average quantity of water supplied daily by this company, during the twelve months ending 31st Dec. 1853, was 11,990,989 gallons. This supply is in course of increase.

### West Middlesex Waterworks.

The average quantity of water supplied daily by the West Middlesex Waterworks, for seven days in the week, during the twelve months ending 31st Dec. 1853, was 5,000,606 gallons. This company will shortly be enabled to increase their supply of water by about 6,000,000 gallons per diem.

### Grand Junction Waterworks.

The average quantity of water actually pumped daily for six days in each week, from the 1st of January to the 31st of December 1853, amounted to 5,968,288 imperial gallons, equivalent to a daily supply delivered for each of the 365 days during the year of 5,115,675 imperial gallons. The number of houses, manufactories, and public establishments supplied with water to the same period, amounts to 16,019. Upon the completion of the company's new works, they will have the power of increasing their supply to above 20,000,000 imperial gallons per diem.

### Southwark and Vauxhall Water Company.

Quantity of Water supplied by the Company, from the 1st January 1853 to the 31st December 1853.

| | Gallons. |
|---|---|
| Total quantity pumped in the year . . . | 2,652,573,277 |
| Being an average per week of . . . . | 51,011,024 |
| Or an average per day (for six days in the week) | 8,501,837 |

This supply can be more than doubled if required; the 36-inch main, with the engine-power provided at Hampton, will deliver at Battersea 20,000,000 gallons in the twenty-four hours.

### Lambeth Waterworks.

The average quantity of water delivered daily by the Lambeth Waterworks Company, in the twelve months ending 31st December 1853, was 5,603,000 gallons per diem. The supply can be increased from the existing works of this company at Seething Wells to upwards of 10,000,000 gallons per day.

### Chelsea Waterworks.

The average daily snpply of water by the Chelsea Waterworks Company, during the twelve months ending 31st December 1853, was 5,632,000 imperial gallons. The supply of water can be increased about 15 per cent. from the present works, and new works are in course of construction above Kingston-on-Thames, under the Chelsea Waterworks Act, 1852, which will enable the company to double the quantity.

### Kent Waterworks.

The total quantity delivered was 671,942,164 gallons. The average daily supply of six days per week was 2,146,780 gallons. The foregoing is the year's and average daily supply delivered by the company's engines during 1853; but in estimating the power of supply of a waterworks, it should be taken at the highest day's demand. In the course of last summer, this has risen frequently at these works to near 4,000,000 gallons, and could without any inconvenience be increased to any quantity required by the district.

### Hampstead Waterworks.

During the year 1853, the number of houses supplied by the Hampstead Waterworks Company was 5,454. The average daily supply of water was 607,368 gallons; of this quantity about 3,000 gallons per day were for manufacturing purposes. During the six months, from March to October, an additional quantity, averaging 45,000 gallons a day, was supplied for the purpose of watering streets and roads. There is no public establishment in the district supplied by these works. For the purpose of increasing and improving their resources, this company are boring through the chalk at their works in Kentish Town, in the expectation of deriving an abundant supply of pure water from the green sand. The depth of the boring is now 940 feet. The work is prosecuted with all possible speed, and the directors have every reason to think it will soon be brought to a successful issue. Meantime, arrangements have been made with the New River and West Middlesex Waterworks, and the mains connected, so as to furnish any quantity of water which may be required in the district.

These returns are most valuable, as shewing the enormous volume of water poured daily into London, and the facilities possessed by the water companies for ensuring and even increasing the supply. The main proportion of this water is derived from the Thames. Suggestions have often been made to supply the Great Metropolis from other sources. The last proposal of this kind is to the effect of obtaining water for at least a part of London from the river Colne. The proposer believes that the pure water thus obtained could be delivered at Kilburn 120 feet above the datum of the Ordnance map, owing to which it could be supplied at high service to all houses in Piccadilly, and between that street and the Thames, including of course all the suburbs, by mere gravitation. The realisation of this plan would unquestionably be of moment. It would seem, however, that the Thames, after all, is the only safe source of supply as regards quantity, and that a more correct application of the engineer's and chemist's knowledge would speedily render this supply equally safe in respect to quality.

---

